# The impact of nurse-led, AI-assisted perioperative health education on psychological status and quality of life in patients undergoing lung cancer surgery

**DOI:** 10.3389/fpsyg.2025.1702256

**Published:** 2025-11-27

**Authors:** Chenminghuang Shen, Yuzhu Huang, Bingbing Wang, Xiaoyan Ye, Jinzhi Jiang

**Affiliations:** 1 Department of Thoracic Surgery, The First Affiliated Hospital of Xiamen University, Xiamen University, Xiamen, Fujian, China; 2 Department of Gynaecology and Obstetrics, The First Affiliated Hospital of Xiamen University, Xiamen University, Xiamen, Fujian, China; 3 Department of Neonatology, The First Affiliated Hospital of Xiamen University Xiamen University, Xiamen, Fujian, China

**Keywords:** AI-assisted education, perioperative care, lung cancer, quality of life, psychological status

## Abstract

**Objective:**

To evaluate the clinical value of integrating artificial intelligence (AI) into perioperative health education for patients undergoing surgery for lung cancer.

**Methods:**

This retrospective study included patients who underwent radical resection for non-small cell lung cancer (NSCLC) in the Department of Thoracic Surgery at the First Affiliated Hospital of Xiamen University between January and December 2024. According to the perioperative education method, patients who met the inclusion criteria from January to May 2024 were included in the conventional group, whereas those from June to December 2024 were included in the AI-assisted group. All patients received standardized rehabilitation education based on a unified handbook. On this basis, the AI-assisted group additionally received individualized health education and psychological counseling generated by AI, which was verified and supplemented by nurses. Clinical data and validated questionnaire scores were collected and analyzed.

**Results:**

A total of 135 patients were included, with 70 in the conventional group and 65 in the AI-assisted group. There were no significant differences in demographic and clinical characteristics between the groups. Compared with the conventional group, patients in the AI-assisted group scored significantly higher on the “confrontation” dimension of the Medical Coping Modes Questionnaire (MCMQ), indicating a stronger tendency toward active coping, whereas no significant differences were observed in the “avoidance” and “acceptance–resignation” dimensions. Regarding psychological status, the AI-assisted group had significantly lower depression and anxiety scores postoperatively, while stress scores showed no significant difference. Quality of life, assessed by the World Health Organization Quality of Life-BREF (WHOQOL-BREF), revealed that the AI-assisted group had significantly higher scores in the psychological and social domains as well as the overall score, with no significant differences in the physical or environmental domains.

**Conclusion:**

Nurse-led perioperative health education supported by AI tools can help optimize patients' coping strategies, reduce negative psychological states, and improve quality of life after lung cancer surgery.

## Introduction

Lung cancer remains the leading cause of cancer-related morbidity and mortality worldwide, posing a substantial threat to public health (Siegel et al., 2022). Non-small cell lung cancer (NSCLC) accounts for approximately 85% of all lung cancer cases, and surgery remains the primary treatment option for patients diagnosed at an early stage (Leiter et al., 2023). The perioperative period is not only a critical stage of surgical treatment but also a time when patients often experience heightened psychological stress and a marked decline in quality of life (Ma et al., 2024). Previous studies have demonstrated that most lung cancer patients experience varying degrees of anxiety and depression during the perioperative period, which may reduce their trust in medical care, compromise treatment adherence, and ultimately affect postoperative recovery and long-term prognosis (Gao et al., 2022; Papaconstantinou et al., 2025). At the same time, quality of life has been widely recognized as an important indicator for evaluating the overall effectiveness of cancer treatment, encompassing physical functioning, psychological wellbeing, and social adaptation, all of which are directly linked to the patient's overall treatment experience and rehabilitation outcomes (Hopwood and Stephens, 2000).

Conventional perioperative health education is typically delivered by nurses or physicians in a unidirectional manner, which lacks sufficient interaction and personalization, and is often associated with a high rate of information loss. Some patients attempt to supplement their knowledge through the internet or social media, but the reliability of such information is questionable and may inadvertently exacerbate psychological distress (Rafieinasab et al., 2024). Thus, providing effective and individualized health education and psychological support during the perioperative period has become a pressing issue in perioperative nursing practice. In recent years, artificial intelligence (AI) has been increasingly applied in medical education and health management. Large language model-based AI tools can deliver personalized explanations and interactive question-and-answer sessions tailored to each patient's condition, combining both standardization and flexibility. Beyond providing information, AI-assisted education may enhance patient engagement and self-efficacy, enabling patients to actively participate in their recovery and feel more confident in managing their condition. Furthermore, by providing consistent and comprehensible explanations, AI tools can reduce uncertainty, a key psychological stressor during the perioperative period, thereby improving emotional wellbeing and adaptive coping. Previous studies have shown that AI-assisted health education significantly alleviates perioperative anxiety and enhances satisfaction with surgical informed consent in patients undergoing total knee arthroplasty (Gan et al., 2025). In the field of breast cancer rehabilitation, digital platforms combined with the Teach-Back method have also been shown to improve quality of life and reduce negative emotions (Ahmadidarrehsima et al., 2020). However, research on the application of AI-assisted health education in the perioperative care of patients undergoing lung cancer surgery remains limited. Therefore, the present study aimed to evaluate the impact of AI-assisted perioperative health education on the psychological status and quality of life of patients undergoing lung cancer surgery. By comparing AI-assisted and conventional health education approaches, this study seeks to provide new evidence for optimizing perioperative nursing models in thoracic surgery and improving patients' overall treatment experience.

## Methods

This study was an observational study comparing the effects of AI-assisted perioperative health education and conventional education on psychological status and quality of life in patients undergoing lung cancer surgery. The data analysis was conducted retrospectively. This study included patients who underwent thoracoscopic or robot-assisted radical resection for non-small cell lung cancer (NSCLC) in the Department of Thoracic Surgery at the First Affiliated Hospital of Xiamen University between January and December 2024. Patients were included in different groups according to the perioperative health education strategies implemented during different time periods. This study represents a retrospective analysis based on the institutional adjustment of health education practices. Except for differences in the educational approach, all surgical procedures, nursing workflows, and perioperative management standards remained consistent throughout the study period. Patients who received conventional education between January and May 2024 were included in the conventional group, whereas those who received conventional education supplemented with AI-assisted education between June and December 2024 were included in the AI-assisted group. The study protocol was approved by the institutional ethics committee, with a waiver of written informed consent.

Inclusion criteria were: (1) pathologically confirmed NSCLC and completion of radical surgery; (2) participation in postoperative pulmonary rehabilitation, including breathing exercises, effective coughing, and physical rehabilitation training; and (3) intact cognitive function with the ability to understand educational content and adhere to rehabilitation plans. Exclusion criteria were: (1) severe chronic pulmonary disease that could compromise rehabilitation outcomes; (2) major postoperative complications leading to interruption of rehabilitation; (3) incomplete key data required for this study.

### Perioperative health education

Both groups received standardized education based on the Thoracoscopic Lung Cancer Postoperative Rehabilitation Training Handbook, which covered five domains: (1) exercise training, including aerobic and strength training; (2) breathing training, including diaphragmatic breathing, pursed-lip breathing, and balloon-blowing exercises; (3) dietary and nutritional guidance; (4) psychological counseling to relieve perioperative anxiety and depression; and (5) smoking cessation guidance to promote long-term recovery. In the conventional group, nurses delivered face-to-face education, while physicians or nurses addressed patients' disease-related concerns and psychological issues during the perioperative period. The duration of each perioperative health education session was approximately 25–30 min for both groups. The AI-assisted group used AI-generated materials for personalized instruction, but the total time per session did not differ substantially from the conventional group. The educational content included the same core topics (disease knowledge, surgical procedures, rehabilitation, pain management, and psychological support), with the AI system providing individualized expressions and examples.

In the AI-assisted group, ChatGPT-4 was integrated into routine education. Educational content was tailored to each patient based on their medical history, educational background, cognitive ability, and pulmonary function. After the education session, the AI posed key questions to prompt patients to restate core information, thereby assessing comprehension. If errors were identified, the system provided simplified explanations or mnemonic prompts to reinforce learning and reduce misunderstanding. In addition, ChatGPT-4 addressed disease-related questions and psychological concerns raised by patients during the perioperative period, generating clear and comprehensible explanations to mitigate uncertainty-related anxiety. For example, when a patient expressed concern about postoperative chest pain, the AI provided an explanation regarding typical pain patterns after lobectomy and guided the patient through relaxation breathing exercises. In another instance, it prompted patients to restate key steps of breathing training to ensure comprehension.

All AI-generated responses were reviewed by the nurse during the same session before being presented to the patient. Nurses checked for medical accuracy, linguistic appropriateness, consistency with clinical guidelines, and alignment with each patient's condition, and provided additional explanations when necessary. The supplementary modifications mainly involved adjusting medical terminology, refining psychological guidance language, or adding individualized examples. Approximately 10%−20% of AI-generated content required such supplementation, and the entire verification process typically required about 5–10 min per patient.

### AI model configuration

ChatGPT-4 was accessed via the OpenAI API through a local workstation. No plugins or conversation history functions were enabled. The model parameters were set to a temperature of 0.7 and a top-*P* value of 0.9. Each patient inquiry was processed in an independent session to ensure accuracy and consistency.

### Data collection

#### Medical Coping Modes Questionnaire (MCMQ)

The Medical Coping Modes Questionnaire (MCMQ), originally developed by Feifel et al. (1987) and widely used to assess coping styles, consists of three dimensions: confrontation, avoidance, and acceptance–resignation. It employs a four-point Likert scale ranging from “never” to “often,” with higher scores indicating a greater tendency toward that coping style.

#### Depression Anxiety Stress Scale-21 (DASS-21)

The Depression Anxiety Stress Scale-21 (DASS-21), a validated Chinese version of the abbreviated 21-item scale (Wang et al., 2016), was used to assess depression, anxiety, and stress. It consists of 21 items, with seven items per subscale. Each item is rated on a four-point Likert scale (0–3), with higher scores indicating greater symptom severity. Subscale scores range from 0 to 21, with a total score range of 0 to 63. The DASS-21 was used to measure depression, anxiety, and stress before and after surgery, allowing us to assess the impact of AI-assisted education compared to conventional methods on psychological stat.

#### World Health Organization Quality of Life-BREF (WHOQOL-BREF)

The World Health Organization Quality of Life-BREF (WHOQOL-BREF), validated in the Chinese population (Chang et al., 2022), is a 26-item short form of the WHOQOL-100. It evaluates four domains—physical health, psychological health, social relationships, and environment—using a five-point Likert scale (1 = strongly agree to 5 = strongly disagree). Higher scores reflect better health-related quality of life (HRQOL).

Clinical data were extracted from the electronic medical record and nursing information systems, including age, gender, body mass index (BMI), smoking history, education level, clinical staging, surgical types, and length of hospital stay. Scale scores from the MCMQ, DASS-21, and WHOQOL-BREF were also recorded. The second assessment was conducted on postoperative day 2. Data collection was conducted by trained research assistants, with strict adherence to ethical and privacy protection standards.

### Statistical analysis

Continuous variables were expressed as mean ± standard deviation or median (interquartile range), and categorical variables as frequency and percentage. Group comparisons were performed using independent-sample *t*-tests for normally distributed continuous variables, Mann–Whitney *U*-tests for non-normally distributed data, and chi-square tests or Fisher's exact tests for categorical variables. All statistical tests were two-sided, with a significance threshold of *P* < 0.05. Analyses were conducted using IBM SPSS Statistics version 25.

## Results

A total of 135 eligible patients with lung cancer were included in the study, with 70 in the conventional group and 65 in the AI-assisted group. There were no statistically significant differences between the two groups in terms of demographic and clinical characteristics, including age, gender, BMI, smoking history, clinical stage, educational level, surgical type, and length of hospital stay (*P* > 0.05; Table 1).

**Table 1:** Comparison of general data of two groups of patients.

	**Conventional group (*n* = 70)**	**AI-assisted group (*n* = 65)**	***P*-value**
Age (years)	53.5 ± 11.2	55.7 ± 10.3	0.237
Gender (%)			
Male	39 (55.7)	33 (50.8)	0.565
Female	31 (44.3)	32 (49.2)	
BMI (kg/m^2^)	23.2 ± 2.8	23.0 ± 2.7	0.835
Smoking history (%)	32 (45.7)	28 (43.1)	0.758
Clinical staging (%)			
I–II	60 (85.7)	57 (87.7)	0.736
III	10 (14.3)	8 (12.3)	
Educational level (%)			
Primary school and below	11 (15.7)	9 (13.8)	0.847
Junior school	16 (22.9)	13 (20.0)	
Senior high school	28 (40.0)	31 (47.7)	
Bachelor degree or above	15 (21.4)	12 (18.5)	
Surgical types			
Robot-assisted thoracic surgery	9 (12.9)	11 (16.9)	0.506
Video-assisted thoracic surgery	61 (87.1)	54 (83.1)	
Length of hospital stay (days)	7.4 ± 2.2	6.9 ± 1.9	0.263

Comparison of MCMQ scores showed that patients in the AI-assisted group had significantly higher scores in the “confrontation” dimension than those in the conventional group (19.1 ± 4.1 vs. 15.2 ± 3.7, *P* < 0.001), indicating a stronger tendency toward active coping. No significant differences were observed between groups in the “avoidance” or “acceptance–resignation” dimensions (*P* > 0.05; Table 2).

**Table 2:** Comparison of coping styles (MCMQ) between the conventional and AI-assisted groups.

	**Conventional group (*n* = 70)**	**AI-assisted group (*n* = 65)**	**Mean difference (95% CI)**	***P*-value**
Confrontation	15.2 ± 3.7	19.1 ± 4.1	3.9 (2.58, 5.22)	< 0.001
Avoidance	15.9 ± 3.5	14.7 ± 3.0	−1.2 (−2.6, 0.21)	0.118
Acceptance-resignation	9.3 ± 2.1	8.5 ± 1.8	−0.8 (−1.82, 0.16)	0.229

Psychological status, assessed using the DASS-21, revealed no significant differences between groups at admission (*P* > 0.05). At discharge, however, the AI-assisted group demonstrated significantly lower depression scores (8.7 ± 2.5 vs. 10.5 ± 3.6, *P* < 0.001) and anxiety scores (8.5 ± 2.3 vs. 11.4 ± 2.9, *P* < 0.001) compared with the conventional group, while stress scores showed no significant difference (*P* > 0.05; Table 3).

**Table 3:** Comparison of DASS-21 scores at admission and discharge between the two groups.

	**Conventional group (*n* = 70)**	**AI-assisted group (*n* = 65)**	**Mean difference (95% CI)**	***P* value**
**At the time of admission**				
Depression score	6.4 ± 2.1	6.5 ± 1.9	0.1 (−0.57, 0.77)	0.602
Anxiety score	7.7 ± 2.6	7.2 ± 2.9	0.5 (−1.43, 0.43)	0.287
Stress score	6.3 ± 1.8	6.6 ± 1.7	0.3 (−0.29, 0.89)	0.325
**At the time of discharge**				
Depression score	10.5 ± 3.6	8.7 ± 2.5	−1.8 (−2.48, −0.76)	< 0.001
Anxiety score	11.4 ± 2.9	8.5 ± 2.3	−2.9 (−3.78, −2.02)	< 0.001
Stress score	10.7 ± 2.2	10.4 ± 2.1	−0.3 (−1.03, 0.44)	0.422

Regarding quality of life, as measured by the WHOQOL-BREF, the AI-assisted group scored significantly higher in the psychological domain (15.8 ± 3.5 vs. 13.3 ± 3.1, *P* = 0.001), the social relationships domain (16.1 ± 4.7 vs. 14.5 ± 3.6, *P* = 0.027), and the total score (61.6 ± 7.0 vs. 57.8 ± 5.5, *P* = 0.002) compared with the conventional group. No significant differences were found in the physical or environmental domains (*P* > 0.05; Table 4).

**Table 4:** Comparison of WHOQOL-BREF scores between the two groups at the time of discharge.

	**Conventional group (*n* = 70)**	**AI-assisted group (*n* = 65)**	**Mean difference (95% CI)**	***P*-value**
Physiological field	12.5 ± 2.6	13.4 ± 2.7	0.9 (−0.28, 2.08)	0.092
Psychological field	13.3 ± 3.1	15.8 ± 3.5	2.5 (1.38, 3.62)	0.001
Social relations field	14.5 ± 3.6	16.1 ± 4.7	1.6 (0.18, 3.02)	0.027
Environmental field	13.4 ± 3.8	12.2 ± 3.5	−1.2 (−2.47, 0.11)	0.059
Total score of quality of life	57.8 ± 5.5	61.6 ± 7.0	3.8 (1.67, 5.93)	0.002

## Discussion

This study explored the effectiveness of AI–assisted perioperative health education in patients undergoing radical surgery for lung cancer, with a particular focus on medical coping styles, psychological status, and quality of life. The findings demonstrated that compared with conventional health education, AI-assisted education more effectively alleviated perioperative anxiety and depression. Results of the MCMQ indicated that patients who received educational plans developed by profissionals using AI support were more likely to confront their disease, actively seek information, and cooperate with treatment and rehabilitation. Furthermore, quality-of-life assessments revealed that scores in the psychological and social relationship domains were higher in the AI-assisted group than in the conventional group, underscoring the positive role of AI in optimizing psychological wellbeing and quality of life during the perioperative period.

The present results revealed that patients in the AI-assisted group scored significantly higher on the “confrontation” dimension of the MCMQ, indicating that nurse-led education supported by AI enhanced patients' understanding and use of adaptive coping mechanisms, particularly confrontive coping strategies, to deal with their illness. Previous evidence suggests that confrontation coping is closely associated with improved psychological adjustment and higher quality of life, whereas avoidance and acceptance–resignation coping styles are linked to negative psychological outcomes, including anxiety, depression, and poor prognosis (Liu et al., 2025). Through individualized information delivery and interactive communication, AI-assisted education enhanced patients' sense of control and engagement in the treatment process. This positive feedback loop not only alleviated anxiety arising from uncertainty but also facilitated the establishment of healthier coping mechanisms, thereby improving psychological status and overall quality of life (Darabos et al., 2021).

Our study confirmed that patients undergoing radical surgery for lung cancer generally face a high psychological burden, with a substantial proportion experiencing anxiety and depression. These findings are consistent with those of Valentine et al., who reported that perioperative lung cancer patients often experience psychological distress due to uncertainty about surgical risks and long-term prognosis (Yang et al., 2024). Several factors may contribute to this phenomenon: inadequate understanding of surgical resection, anesthesia, and postoperative recovery can lead to fear and tension; the high lethality and recurrence risk of lung cancer further intensify psychological distress; and insufficient family or social support may exacerbate feelings of helplessness and anxiety (Valentine et al., 2024; Jung et al., 2018). Prior studies have also demonstrated that lung cancer patients undergoing surgery experience significantly higher psychological stress both preoperatively and in the early postoperative period compared with the general population, which indirectly supports our results (Chen et al., 2023). In this study, AI-assisted education provided personalized explanations and interactive question–answer sessions, helping patients better understand treatment and recovery processes. This approach reduced uncertainty, mitigated misinformation, and thereby effectively lowered anxiety and depression levels.

Improvements in quality of life were particularly evident in the psychological and social domains among patients in the AI-assisted group. This may be attributed to AI's ability to provide not only disease-related explanations but also emotional support and psychological counseling, which helped reduce feelings of isolation and uncertainty. These findings align with prior studies demonstrating that digital health interventions can improve psychological outcomes and quality of life in cancer patients (Morrison et al., 2017; Sarna et al., 2010). As medical practice increasingly shifts toward a patient-centered model, perioperative care must address not only the prevention of complications and physical recovery but also the enhancement of psychological wellbeing and quality of life. The AI-assisted education model provides a valuable complement to conventional nursing, offering a “AI-assisted + human verification” closed-loop approach that improves the efficiency and accuracy of information delivery while ensuring continuous, interactive, and individualized psychological support.

This study has several limitations. First, as a single-center retrospective study with a relatively small sample size, the findings may be subject to selection and temporal biases, limiting the generalizability of the results. Specifically, since the two groups were included during different periods, there may still be a potential temporal bias, even though our institution maintained consistent clinical practices and treatment principles across both periods. This potential bias may influence the accuracy of the results. Second, the study primarily evaluated short-term outcomes during the perioperative and early postoperative period; the long-term impact of AI-assisted education on psychological status and quality of life remains to be determined. Finally, the inclusion criteria required patients to have adequate cognitive and learning abilities, which may restrict the applicability of this intervention among individuals with lower educational levels or limited acceptance of digital technologies.

## Conclusion

Nurse-led perioperative health education supported by AI tools can help optimize patients' coping strategies, reduce negative psychological states, and improve quality of life after lung cancer surgery.

## Data Availability

The raw data supporting the conclusions of this article will be made available by the authors, without undue reservation.
